# THE EFFECTS OF NEIGHBORHOOD DEPRIVATION AND PERCEIVED NEIGHBORHOOD PROBLEMS ON DEPRESSIVE SYMPTOMS

**DOI:** 10.1093/geroni/igac059.2766

**Published:** 2022-12-20

**Authors:** Jean Choi, Elizabeth Muñoz, Robin Corley, Sally Wadsworth, Chandra Reynolds

**Affiliations:** The University of Texas at Austin, Austin, Texas, United States; The University of Texas at Austin, Austin, Texas, United States; University of Colorado Boulder, Boulder, Colorado, United States; University of Colorado Boulder, Boulder, Colorado, United States; University of California, Riverside, Riverside, California, United States

## Abstract

The progression of detrimental mental health outcomes may begin before old age, and investigating correlates earlier in the lifespan may provide insights into prevention efforts. Neighborhoods have been linked with mental health in adulthood, but there is variation in measurement across studies. Studies have examined the independent associations between either of objective or subjective measures of neighborhoods, but few to none have examined both of these in one study. We assessed the effects of both objective (Area Deprivation Index; ADI) and subjective, or perceived, neighborhood characteristics on depressive symptoms among early midlife adults, using data from the Colorado Adoption/Twin Study of Lifespan behavioral development and cognitive aging (Nf1166, Mage=33.51 (5.07)). Participants completed self-report assessments of perceptions of their neighborhoods (e.g., perceived safety, disorder). Addresses were geocoded and linked with 2010 Census data, and we created a standardized ADI composite. To account for subject non-independence, we fitted multilevel linear regressions, controlling for relevant confounding variables. Results showed that higher ADI was linked with greater depressive symptoms (d = .04), but the effect of ADI was no longer significant and was reduced by about 30% after accounting for subjective neighborhood domains. Perceived neighborhood disorder had the largest effect (d = .19) and remained the sole predictor of depressive symptoms when all perceived domains were included in one model. Findings suggest that residents’ subjective interpretations of their neighborhood surroundings may be shaped by the broader structural contexts in which they live in, revealing a potential pathway through which objective neighborhoods affect depression.

